# Mixed-Type Carotid Sinus Syndrome in a Patient With Advanced Laryngeal Cancer: A Case Report

**DOI:** 10.1155/carm/6611725

**Published:** 2025-10-03

**Authors:** Montaser Elkholy, Naisargee Solanki, Mohamed Abdelazeem, Zijin Lin, Yasemin Bahar, Zain U. L. Abideen Asad, M. Chadi Alraies

**Affiliations:** ^1^Department of Medicine, Detroit Medical Center, Wayne State University, Detroit, Michigan, USA; ^2^Cardiovascular Department, UMass Chan-Baystate Medical Center, Springfield, Massachusetts, USA; ^3^College of Osteopathic Medicine, Michigan State University, East Lansing, Michigan, USA; ^4^Department of Medicine, University of Oklahoma Health Sciences Center, Oklahoma City, Oklahoma, USA; ^5^Cardiovascular Institute, Detroit Medical Center, Detroit, Michigan, USA

**Keywords:** bradycardia, cancer, treatment

## Abstract

Head and neck tumors can rarely cause carotid sinus syndrome (CSS), a condition characterized by bradycardia, hypotension, and syncope. A 68-year-old male with advanced laryngeal cancer presented with syncope. Examination revealed a fixed 5 cm submandibular mass. Computed tomography angiography (CTA) neck showed a large mass encasing the carotid arteries. During the hospital stay, the patient experienced recurrent bradycardia and hypotension, which were resolved when his neck was turned to the left. Diagnosis of mixed-subtype CSS secondary to tumor compression was assumed. Blood tests, EKG, and CT imaging ruled out other causes. The tumor's encasement of the carotid arteries likely triggered the carotid sinus reflex during head movement. Both cardioinhibitory and vasodepressor components were present, suggesting a mixed subtype of CSS. CSS should be considered in patients with head and neck cancer, presenting with unexplained bradycardia or hypotension. Multidisciplinary management is the key to accurate diagnosis and treatment.

## 1. Introduction

**Figure 1:**
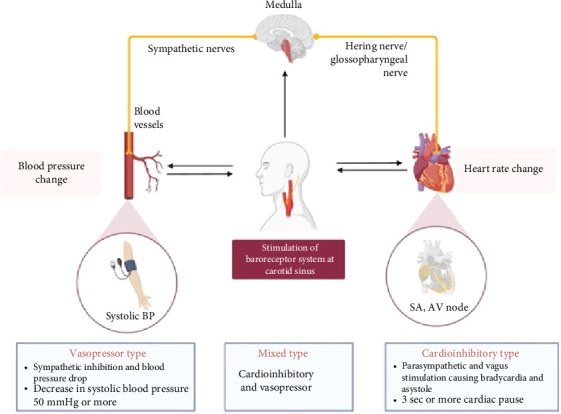
Pathophysiology and types of carotid sinus syndrome.

An important regulatory mechanism for maintaining arterial blood pressure (BP) in the body is the carotid sinus reflex. The baroreceptor system, which regulates BP and maintains homeostasis, is integrated by the carotid sinus and the aortic arch bodies; it can be felt at the level of the thyroid cartilage, right below the angle of the jaw [1]. The carotid sinus's tunica adventitia contains the receptors for the carotid sinus reflex. These receptors produce impulses in response to stretching of the artery wall, which are then sent to the medulla by the Hering nerve (via the glossopharyngeal nerve). The sinoatrial (SA) node, the atrioventricular (AV) node, and other blood arteries in the body are all impacted by the efferent fibers' impulse transmission through sympathetic adrenergic neurons and the vagus nerve [1] (Figure 1). Parry observed in 1799 that placing pressure on one of the carotid sinuses can slow down the pulse rate [2]. Carotid sinus hypersensitivity (CSH) is the term for an amplification of the response to this maneuver. The majority of CSH patients have no symptoms. However, CSH might occasionally present as cerebral ischemia, hypotension, syncope, or spontaneous dizziness; this condition is known as carotid sinus syndrome (CSS).

The 2017 American College of Cardiology/American Heart Association/Heart Rhythm Society (ACC/AHA/HRS) and the 2018 European Society of Cardiology (ESC) syncope guidelines provide the most widely accepted definition of CSH, which is defined as heart rate (HR) pauses longer than three seconds and a systolic BP drop greater than 50 mmHg [3, 4]. These values characterize the primary subtypes of CSS: vasodepressor (5%–10%), cardioinhibitory (70%–75%), and mixed type (20%–25%); in which there is a decrease in HR and vasomotor tone (Figure 1).

The incidence of CSS increases with advancing age and has a strong male dominance (> 2:1). It accounts for 1% of syncope cases, and head and neck tumors can occasionally lead to CSS [5]. We present a case of advanced laryngeal carcinoma presenting with syncope and intermittent episodes of bradycardia and hypotension secondary to CSS [6].

## 2. Case Presentation

A 68-year-old male with a past medical history of Stage 4 laryngeal squamous carcinoma, cerebrovascular stroke with residual right-sided deficits, hypertension, and dyslipidemia presented to the hospital with syncope. He reported abrupt episodes of dizziness and blurred vision followed by loss of consciousness, without any symptoms of palpitations, dyspnea, or chest pain. These episodes typically lasted a few seconds with full recovery within a few minutes.

Four months prior to admission, the patient underwent nasopharyngolaryngoscopy, which showed a right arytenoid erythematous enlarged mass as well as a fixed and immobile right vocal cord; the left aryepiglottic fold had an ulcerative lesion and a large right neck cervical mass measuring 3 × 3 cm, which was immobile anterior to the right sternocleidomastoid muscle. Biopsy of the right laryngeal mass and left laryngeal mass showed invasive squamous cell carcinoma and poorly differentiated squamous cell carcinoma in situ.

**Figure 2:**
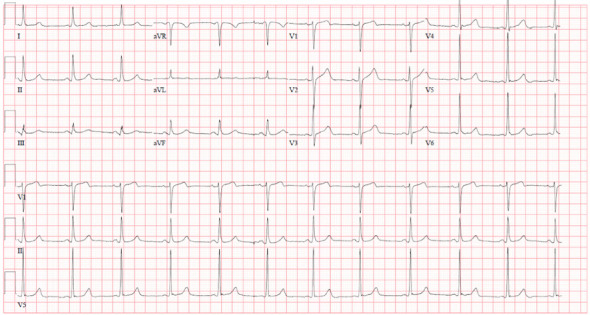
12-lead EKG demonstrating normal sinus rhythm without abnormalities.

**Figure 3:**
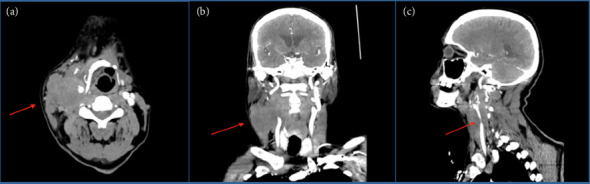
CTA of the neck: (a) transverse view, (b) coronal view, and (c) sagittal view. Red arrows indicate tumor encasement of the right carotid artery.

On admission, his vitals were temperature 36.9°C, HR 95/min, BP 101/70 mmHg, respiratory rate 20/min, and saturation at 99% on room air. On physical examination, the patient had a palpable 5 cm mass in the right submandibular region that was firm and fixed. A routine blood test and measurement of electrolytes, glucose, liver, kidney, thyroid function, and high-sensitivity troponin T were all negative. EKG showed normal sinus rhythm (Figure 2). CT head showed encephalomalacia in the left thalamus as a sequela of remote hemorrhage and no evidence of acute intracranial abnormality. CT angiogram (CTA) of cerebral arteries demonstrates normal contrast enhancement of the major intracranial arteries without occlusion, stenosis, or filling defects and symmetric distribution of the distal arterial branches without areas of abnormal vascularity. The CTA neck showed a large heterogeneously enhancing mass in the right lateral neck extending from the clavicle to the supraclavicular region measuring 6.6 × 5.5 × 8.4 cm. The mass encases the right common and internal carotid arteries (Figure 3).

During the hospital course, the patient developed attacks of hypotension and bradycardia, followed by loss of muscle tone and consciousness with full recovery in a few minutes after a normal saline bolus. No specific triggers were apparent. Repeated head CT showed no acute intracranial abnormality. We noticed that during lesion and large these attacks when we turned the patient's neck to the left (opposite side of the lesion), the HR returned to normal. The patient did not complain of chest pain, dyspnea, or palpitations. Cardiac biomarkers and electrolytes were normal. Transthoracic echocardiography (TTE) showed no abnormal findings. A diagnosis of CSS from tumor compression was suspected.

The management plan for the patient focused on targeting the tumor itself, with the initiation of radiotherapy. However, due to CTA findings revealing encasement and compression of the common and internal carotid arteries by the tumor, the neuroendovascular surgery team was consulted, and carotid stenting was performed. The patient was subsequently transferred to the neurocritical care unit for recovery. On the morning of postoperative Day 1, the patient experienced sudden pulseless electrical activity, and CPR was attempted but was ultimately unsuccessful.

## 3. Discussion

The CSS is the set of symptoms and signs resulting from an exaggerated response of the carotid sinus reflex. The 2017 ACC/AHA/HRS and the 2018 ESC syncope guidelines provide the most widely accepted definition of CSH, which is defined as HR pauses longer than three seconds and a systolic BP drop greater than 50 mmHg [3, 4]. These values characterize the primary subtypes of CSS: vasodepressor (5%–10%), whose primary symptom is a vasomotor tone decrease without a decreased HR; cardioinhibitory (70%–75%), which results in sinus bradycardia; AV block, or asystole as a result of vagal action on the SA and AV nodes; and mixed type (20%–25%), in which there is a decrease in HR and vasomotor tone.

The majority of CSS cases have no clear triggering factor. The known trigger mechanisms include movement of the head and neck, hyperextension, wearing tight neckwear (such as collars), and shaving [7]. CSS can also result from any pathology near the carotid sinus, including internal carotid artery aneurysms, neck malignancies, and carotid body tumors [5, 8].

Weiss and Baker reported the first case of CSS associated with head and neck cancer in 1933 [9]. Strong carotid sinus response in cases of neck malignancies is explained by the tumor pressing on the carotid body or by the tumor invading the carotid sinus or glossopharyngeal nerve [5]. The carotid sinus's tunica adventitia contains the receptors for the carotid sinus reflex. These receptors produce impulses in response to stretching of the artery wall, which are then sent to the medulla by the Hering nerve (via the glossopharyngeal nerve). BP and HR drop as a result of interneurons' activation of the efferent pathways, which stimulates the parasympathetic nervous system and inhibits the sympathetic nervous system [1].

There have been rare, documented cases of CSS associated with head and neck cancer in the literature, and these cases comprised a cardioinhibitory or vasodepressor response [10]. To our knowledge, our case is the first one with mixed cardioinhibitory and vasodepressor response.

The probable cause of CSS in our patient was the significant tumor encasing the right common carotid artery. Head motions could be the initiating part. We noticed that the patient's HR reverted to normal during bradycardia episodes when we rotated the neck to the left, which is the opposite side of the lesion. We did not do a carotid sinus massage on our patient due to the risk of carotid plaque embolization and the patient's past history of stroke. The most recent guidelines, ESC, advise against carotid sinus massage in order to reduce neurological problems in individuals who have had a stroke in the past [4].

Individuals diagnosed with any type of CSS share the same clinical features: syncope, unexplained falls, and dizziness. It is remarkable that the right and left carotid sinuses respond differently to stimulation. Sigler's study with 345 healthy individuals revealed that the left side is more sensitive to stimulation and is frequently linked to high-grade AV block (Mobitz II and third degree), while the right side typically results in sinus bradycardia [11]. Our case is concordant with Sigler's study as the patient had recurrent episodes of sinus bradycardia and a right-sided neck mass.

The best course of treatment for syncope caused by head and neck malignancies is still under debate. Depending on the type of CSS, two primary treatments could be used. Patients with a significant or pure cardioinhibitory component are recommended for permanent pacemaker placement (ACC/AHA/HRS Class I indication; ESC Class Ib indication) [3, 4]. However, research indicates that following pacemaker implantation, roughly 50% still experience mild or recurrent symptoms [12]. It is yet unknown how best to treat the vasodepressor or mixed types of CSS because the outcomes are not satisfactory. Since cardiac pacing can only regulate the bradycardia component of cardioinhibitory CSS and not the low BP, it is useless in pure vasodepressor CSS. Treatment for CSS's vasodepressor component involves anticholinergics, fludrocortisone, midodrine, and other vasopressors, although it is frequently ineffective [13]. Additional approaches for CSS management include cardiac ganglion plexus ablation, artery adventitial stripping, and carotid sinus denervation; however, the effectiveness of these procedures is not well-established [14]. Definitive treatment consists of surgical removal of the tumor. In our case, the safest option was localized radiation because complete surgical resection was not feasible given the N3 stage; since trials have not demonstrated any advantage in older patients [15].

## 4. Conclusion

Unexplained repeated episodes of bradycardia and hypotension in patients with head and neck cancer should notify the physician about the potential for carotid sinus compression, and CSS should be included in the differential. Effective collaboration among internists, cardiologists, and oncologists is essential for accurate diagnosis, effective treatment, and a favorable result.
